# How persistent identifiers can save scientists time

**DOI:** 10.1093/femsle/fny143

**Published:** 2018-06-19

**Authors:** Alice Meadows, Laure Haak

**Affiliations:** ORCID, 10411 Motor City Drive Suite 750, Bethesda, MD 20817, USA

**Keywords:** ORCID, persistent identifiers, research infrastructure, open source, Crossref, digital object identifiers

## Abstract

Research information is useful only if it can be shared—with other researchers, with research organizations (institutions, laboratories, funders and others), and with the wider community. In our digital age, that means sharing information between data systems. Persistent identifiers (PIDs) provide unique keys for people, places and things, which enables accurate mapping of information between these systems and supports the research process by facilitating search, discovery, recognition and collaboration. This article reviews the main PIDs used in research—digital object identifiers for publications, ORCID iDs for researchers, and a proposed new identifier for research organizations—as well as demonstrating how they are being used, and how, in combination, they can increase trust in research and the research infrastructure.

## Teaser

Persistent identifiers enable trusted connections between researchers, their publications and their organizations that ultimately build trust in research, reducing the potential for errors and saving researchers time and hassle.

According to Nature's 2016 jobs survey, researchers spend over one hour out of every 10 on administrative tasks (form filling, reporting, etc)—and that doesn’t include the time spent applying for a grant or submitting a manuscript! Not only do administrative activities of one sort or another take time away from research and teaching activities, the user experience is often frustrating, for example, because the researcher is being asked to key in the same professional data time and time again. Portuguese funder, Fundação para a Ciência e a Tecnologia (FCT), has even created a simulator to calculate how much this duplication of effort costs.

Digital workflow innovations can help, by automating processes and enabling information to be shared between systems. Overleaf, for example, enables authors to easily collaborate, edit and format their articles for publication. Publons allows researchers to keep track of—and get credit for—their review activities. And there are numerous profile systems—open and proprietary—that researchers can use to store and share their professional information: Europe PubMed Central, F1000, and ScienceOpen in the open category, ResearcherID and Scopus ID in the proprietary one, to name a few.

Underpinning these platforms and systems are persistent identifiers (PIDs) for people, places and things. Defined by Wikipedia as ‘a long-lasting reference to a document, file, web page, or other object,’ a persistent identifier is a digital and resolvable reference to a person, a place or a thing. Of course, researchers have been using and referencing PIDs such as ISBNs and ISSNs since long before the Internet. But the transformation to our digital world has depended in large part on PIDs to enable machine readability. Open and non-proprietary PIDs have proven to be valuable in enabling information sharing within and across systems.

Digital object identifiers (DOIs)—open and persistent unique identifiers for research outputs (things), such as articles, books and book chapters, conference proceedings, data sets and more—are probably the most widely known and used research PIDs, with Crossref and DataCite among the most commonly used services. Launched in 2000 as a cooperative effort between publishers, Crossref's original purpose was to enable persistent cross-publisher citation linking in journals. At the time of writing, over 71 million journal articles and 13 million books and book chapters have been assigned a Crossref DOI, and over 733 million citation links have been created between these DOIs. This digital transformation is making possible improved discovery and delivery of research findings.

A newer persistent identifier, but one with which many researchers are arguably more familiar, is the ORCID identifier (iD) for people—more specifically, for anyone involved in research, scholarship or innovation. Launched as a community-led organization in 2012, ORCID’s mission is: ‘to enable transparent and trustworthy connections between researchers, their contributions, and affiliations by providing an identifier for individuals to use with their name as they engage in research, scholarship and innovation activities.’ Like DOIs, ORCID iDs are open and non-proprietary and enable identification, linking and discovery—but of researchers themselves. Many researchers share the same or a similar name; they work or publish under different versions of their name; they change their name; and their name may get transliterated. They may change institution, country, discipline even. But their ORCID iD always remains the same—and it can be used across hundreds of research information systems, from global commercial platforms like Web of Science and open systems like VIVO, to national and local systems such as custom-built repositories. As of March 2018, over 4.5 million researchers globally had registered for an ORCID iD, with around two thirds of those registrations occurring when researchers publish papers describing their work.

And then there are organization identifiers—persistent identifiers for places. Just like person names, organization names can be abbreviated or change over time. Organizations also merge and divide, launch and go out of business. PIDs ensure that organization names can be used in a standard fashion within and across different systems, and they also offer a means to tie names together over time as organization names and ownership changes. There are several proprietary organization identifier companies already in existence, and a community-led organization identifier working group is currently defining the governance principles and technology specifications for an open, independent, non-profit organization identifier registry specifically for use in scholarly communications.

Individually, each of these identifiers are useful, but their value rises exponentially when they are used collectively in digital workflows, where trusted connections between them can be created and easily shared.

Consider the example of a researcher working in a life sciences lab at a European university, who is applying for grant funding. During the application process, she is prompted to provide her ORCID iD. She signs into her ORCID account (using her ORCID, Google, Facebook or institutional credentials), and authorizes the funder system to collect her iD and exchange information with her ORCID record. The funding system can prompt her to import affiliation, publication and prior funding information from her record. And, if the grant is awarded, the funder can put information about it—their name and organization ID, as well as the title, grant ID and date of award—into her ORCID record, noting that the source of the information is the funding organization itself. The researcher can then easily share this information when submitting a paper about the research the funding supported, and when applying for her next grant.

This information exchange not only streamlines the application process for both the researcher and the funder, but also, by using identifiers—for the person, the funder and the grant—ensures that names are shared in a standard way enabling more accurate reporting. These trusted connections between PIDs for researchers, organizations, funders and publishers are critical for open research.

Millions of these trusted connections have already been created, with new types and research activities being added rapidly, including:

- PIDs for research resources, such as national laboratories, large instruments, scientific collections and rare book archives are streamlining the acknowledgment of the resources researchers use to do their work
- PIDs for affiliations, such as education, employment, service and membership enable recognition for a wider range of contributions researchers make in the service of their discipline
- PIDs for grants are making connections between researchers, funding and publications more transparent

There's no doubt that persistent identifiers are, well, persistent! More publishers, funders and other organizations are building PIDs into their research workflows, making it increasingly easy for researchers to share their iD and enable the creation of trusted connections with other PIDs.

All researchers benefit from PIDs. Early career researchers can get in on the ground floor, using PIDs from the start of their career. Registering for an ORCID iD as a student and using it when applying for grants, publishing their research, completing their thesis, joining a professional association or submitting a poster—each creates a connection that can be added to their own PID graph and that of the whole research ecosystem. Wherever they work in the world, whatever their current field of research, and whatever name they use professionally, PIDs ensure that researchers’ contributions are correctly identified, discoverable and recognized.

## Conflict of interest

The authors are both employed by ORCID, Inc, which is a provider of persistent identifiers for researchers and is covered in the article.

